# Distinct effects of adjuvants on B cell responses to protein or polysaccharide antigens contained in glycoconjugate vaccines

**DOI:** 10.3389/fimmu.2025.1574941

**Published:** 2025-08-22

**Authors:** Sonia Budroni, Elisa Faenzi, Simona Tavarini, Chiara Sammicheli, Francesca Buricchi, Gianfranco Volpini, Erica Borgogni, Fabiana Spensieri, Evita Balducci, Bruno Galletti, Maria Rosaria Romano, Ugo D’Oro, Oretta Finco, Monia Bardelli

**Affiliations:** Bacterial Scientific Area, GSK Vaccine, Siena, Italy

**Keywords:** polysaccharide, vaccine, adjuvant, memory B cells, avidity, mice

## Abstract

**Background:**

Protein-polysaccharide conjugate vaccines rely on the induction of T-cell-dependent responses that support germinal center (GC) reactions to potentiate the expansion of antigen-specific memory B-cell (MBC) populations and high-avidity antibody responses. The effects of adjuvants on B-cell and antibody responses are well described for protein antigens but remain largely unexplored for conjugated polysaccharidic antigens.

**Methods:**

We assessed the effects of five adjuvants present in licensed vaccines (AS01, AS03, AS04, and aluminum hydroxide [Alum]) or under clinical evaluation (AS37) on the magnitude and quality of antigen-specific antibody responses and local/systemic B-cell responses. Naive mice received three immunizations of adjuvanted or non-adjuvanted model glycoconjugate vaccine containing *Staphylococcus aureus* (SA) capsular polysaccharide serotypes 5/8 (CP5/8) conjugated to a tetanus toxoid carrier and inactivated SA HlaH35L toxin.

**Results:**

All AS-containing vaccines increased CP5/8-specific antibody titers and B-cell immunity relative to Alum- or non-adjuvanted formulations. After two immunizations, AS03 (α-tocopherol-containing oil-in-water emulsion) most robustly enhanced CP5/8-specific immunity relative to the other adjuvants or no adjuvant. AS03 induced higher responses of high-avidity antibodies persisting for at least 25 weeks post-immunization and greater expansions of populations of splenic GC B cells, mature MBCs in the lymph node or spleen, and long-lived plasma cells in the bone marrow. These effects increased with each immunization, suggesting the presence of avidity maturation and highlighting the role of the carrier in improving the quality of GC reactions. While HlaH35L-specific responses were augmented by each adjuvant, they lacked significant inter-group differences, pointing to profound differences in the adjuvants’ effects on polysaccharide vs. protein antigens in the mice of the present study.

**Conclusion:**

Investigating the antibody quantity and quality and local and systemic B-cell population expansions in a naive model supports our understanding of how different adjuvants shape the response to the tested polysaccharidic antigens.

## 1 Introduction

Vaccines against bacterial pathogens such as *Staphylococcus aureus* (SA) are urgently needed to curb the spread of antimicrobial resistance (AMR) (1, 2). As T-cell-independent antigens, plain carbohydrates do not induce recall responses after sequential vaccinations due to lack of immunological memory induction, particularly in naive populations (3). Carbohydrate-based vaccines have therefore largely been replaced by protein-polysaccharide conjugate (glycoconjugate) vaccines. This platform has been successfully applied in multivalent meningococcal and pneumococcal vaccines and the *Haemophilus influenzae* type b (Hib) vaccine and is considered an important tool to limit the burden of AMR (4, 5). The covalent linking of polysaccharide antigens, such as bacterial capsular polysaccharides (CPs), to a carrier protein introduces B and T-cell epitopes. As CD4^+^ T helper (Th) cells, and particularly T follicular helper (Tfh) cells, control the magnitude and quality of antibody production by B cells, glycoconjugate vaccines can rely on existing CD4 memory and induce humoral responses consisting mainly of immunoglobulin G (IgG).

By potentiating innate immunity, vaccine adjuvants can stimulate the magnitude and diversification of Th cell responses, which in turn potentiate germinal center (GC) reactions. This results in enhanced response of naive B cells and of memory B cells (MBCs; the main cells that differentiate into functional plasma cells upon antigenic recall), leading to affinity-matured durable antibody responses. For protein-based antigens, several Adjuvant System (AS) families, containing oil-in-water (o/w) emulsions or Toll-like receptor (TLR) agonists, have been developed, which have been shown to enhance the breadth, Fc-mediated features, avidity, and longevity of the antibody response in humans (6–10). Currently, AS01 (MPL and the saponin QS21 in a liposomal formulation), AS03 (o/w emulsion containing α-tocopherol), and AS04 (TLR4 agonist MPL adsorbed to Alum) are present in licensed (protein-based) vaccines (10–12), while AS37 [Alum-adsorbed TLR7 agonist (11, 13, 14)] is currently under Phase I/IIa evaluation.

For carbohydrate-based vaccines, aluminum salts (Alum) are still the only authorized adjuvants (15). While having an excellent safety record, this classical adjuvant is inconsistently effective in this context, as illustrated by the lack of immune enhancements to certain *Shigella* or pneumococcal candidate vaccines in adults and toddlers, respectively (16, 17). Though the precise effects of alternative (non-Alum) adjuvants on glycoconjugates are incompletely understood, there is some preclinical evidence that o/w emulsions or the TLR-based adjuvants CpG, AS04, and AS37 might improve the glycoconjugate vaccine response over Alum alone (4, 15, 18–21). Furthermore, trends of improved human immune responses were observed for AS03, which enhanced the functional antibody response to one antigen of a four-component SA glycoconjugate vaccine compared to no adjuvant (22). In addition, AS37 seemed to induce higher *Neisseria meningitidis* group C (MenC)-specific antibody responses relative to Alum only (11, 14). However, in both studies, demonstrating the added value of using an AS proved challenging due to pre-existing immunity to the vaccine antigen(s) in the participants. A naive immune setting will allow a better appreciation of how these adjuvants work in this context, and we hypothesize that the stronger innate immunity and CD4^+^ T-cell help induced by an AS-containing vaccine will lead to quantitatively and qualitatively enhanced antibody and B-cell responses compared to the Alum- or non-adjuvanted formulations.

As comparative AS studies—as done for protein antigens in animal models and humans (6–9, 23–27)—have not been reported for glycoconjugates, we investigated the qualitative and quantitative effects of AS01, AS03, AS04, AS37, and Alum on the humoral and B-cell responses induced by repeated immunizations with a glycoconjugate vaccine in naïve mice. The three-component model antigen consisted of SA CP serotypes 5 and 8 conjugated to a tetanus toxoid carrier (CP5-TT/CP8-TT) and an inactivated mutant of the SA α-hemolysin protein [‘Hla’ (28)], each of which was also part of the clinically tested four-component SA antigen (22). Effects on antibody titers, antibody avidity, activation of GC reactions, and the local or systemic B-cell expansion and persistence in lymphoid tissues were characterized for the CP5/8-specific responses as well as for the Hla-specific responses serving as an internal protein antigen control.

We found that all five adjuvants enhanced the antibody and primary B-cell responses specific to each antigen. After the antigen recall, however, AS03, an adjuvant present in approved protein-based (pre)pandemic vaccines (12), mediated stronger effects on both the avidity maturation of anti-CP antibodies and the numbers of CP-specific MBCs, suggesting that these effects were linked. We also detected profound differences in the adjuvants’ effects between the tested polysaccharide-based and protein-based antigens. This information can support the adjuvant selection for these glycoconjugates, which could ultimately inform vaccine development based on these challenging but crucially important antigens.

## 2 Materials and methods

### 2.1 Studies and ethics

Before investigating the effects of the five adjuvants on the antibody responses in the main study, we performed adjuvant dose-ranging experiments to identify the optimal adjuvant doses for the induction of anti-CP5/8 IgG responses and evaluated the proof of concept of the B-cell assessments in a preliminary study (see **Supplementary Methods** for details). All studies were performed in GSK’s AAALAC-accredited animal facilities in Siena, Italy.

Husbandry and experiments were ethically reviewed and performed in accordance with Italian and European laws, guidelines, and policies for animal experimentation, housing, and care (Italian D. L. no. 26/14 and the European Directive 2010/63/EU) and GSK’s Policy on the Care, Welfare, and Treatment of Animals. Protocols for all studies were approved by the local ethical review committees of GSK (reference AWB2015-01).

### 2.2 Vaccines

The SA glycoconjugate vaccine antigen contained the mutant inactivated form of SA-Hla toxin (HlaH35L; elsewhere in this article referred to as Hla) and the SA CP5 and CP8 conjugated with tetanus toxoid (CP5-TT and CP8-TT, respectively). Hla was used at 1.38 mg/mL in KH_2_PO_4–_10 mM buffer (pH 7.2). CP5-TT and CP8-TT were used at 0.444 mg/mL and 0.385 mg/mL, respectively (both saccharide-based) in 50 mM NaCl. The injections contained 10 μg Hla and 2 μg each of CP5-TT and CP8-TT in an injected volume of 50 μL (25 μL/leg). Just prior to immunization, the lyophilized antigen was reconstituted with either AS01, AS03, AS04, AS37, Alum (aluminum hydroxide), or phosphate-buffered saline (PBS). One dose of AS01 used in this study contained 2.5 µg each of MPL and QS-21 in a liposome-based formulation. AS03 (25 µL) and the antigen (25 µL) were mixed together 1:1 prior to injection. One dose of AS04 used in this study contained 10 μg MPL adsorbed on 50 μg Al^3+^ in the form of aluminum hydroxide. AS37 used in this study contained 1 µg of the synthetic TLR7 agonist adsorbed on 100 μg Al^3+^ in the form of aluminum hydroxide. One dose of Alum used in this study contained 100 μg Al(OH)_3_.

### 2.3 Animals and immunizations

**Figure 1:**
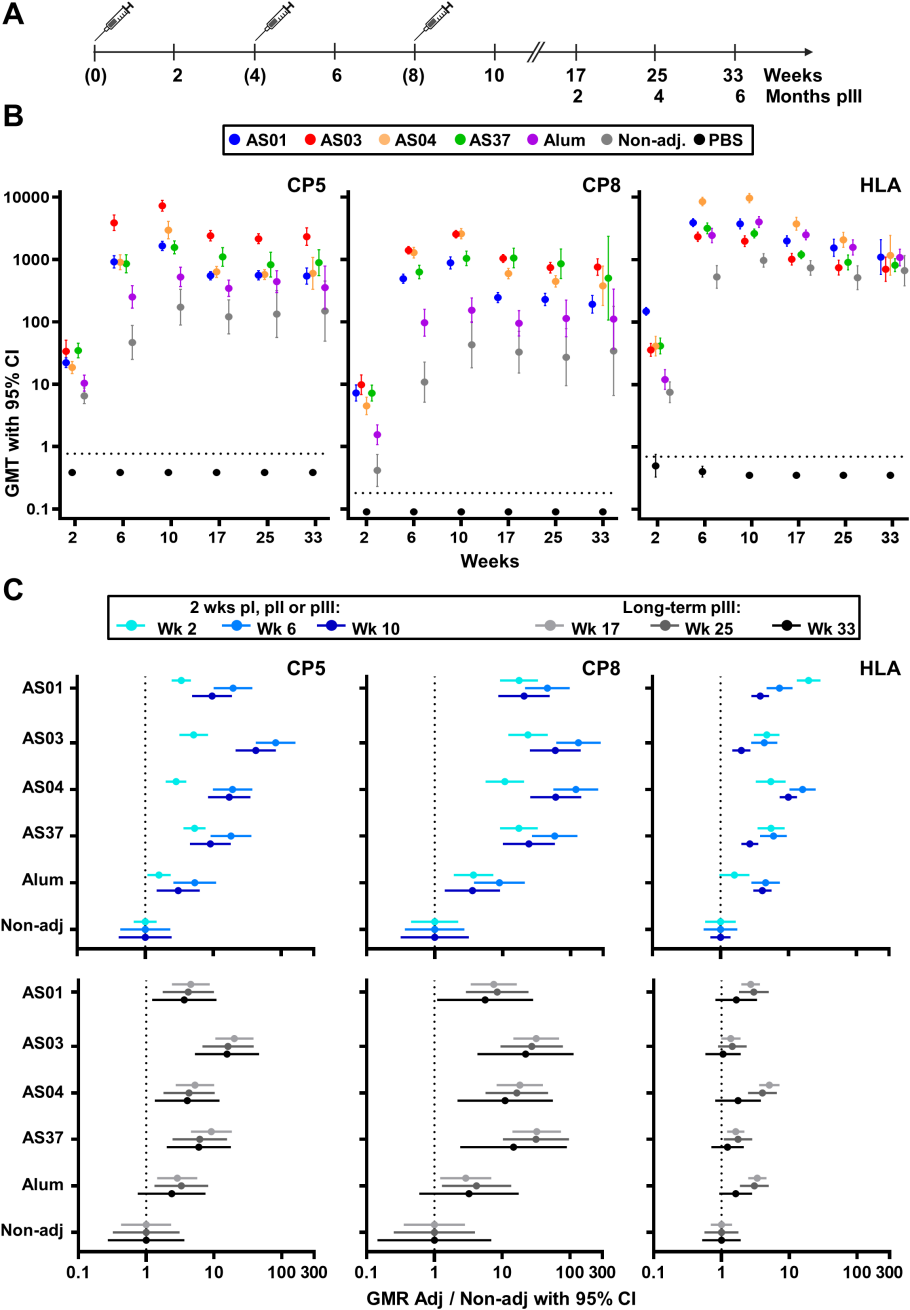
Antibody titers. **(A)** Schedule shows the timepoints of immunization (bracketed) and blood collection for serology assessments. Balb/c mice received three intramuscular injections with vaccines containing a *Staphylococcus aureus* model antigen comprising tetanus-toxoid-conjugated capsular polysaccharide serotypes 5 and 8 (CP5 and CP8, respectively) and HlaH35L (Hla) protein. Vaccines were non-adjuvanted or adjuvanted with AS01, AS03, AS04, AS37, or Alum. A group of phosphate-buffered saline (PBS)-injected mice was used as control. Anti-CP8, anti-CP5 and anti-Hla IgG levels were measured up to week 10 in sera from 16 mice/group, and at 9, 17, and 25 weeks post-dose 3 in sera from 15, 10, and 5 mice/group, respectively. **(B)** Geometric mean titers (GMT) with 95% confidence intervals (CI) are presented as closed circles with vertical bars, color-coded by group as shown in the key. Horizontal dashed lines represent the lower limit of quantification of the assay. **(C)** Geometric mean ratios (GMR) of each adjuvant group over the non-adjuvanted (Non-adj) group are shown with 95% CI for the 2-week timepoints after each dose, and for the three persistence timepoints post-dose 3.

In the main study, 283 5-week-old female BALB/c mice (SPF animals supplied by Charles River) were housed in an individually ventilated cage system and provided with enrichment and unlimited access to food and autoclaved tap water. They were randomly segregated into the vaccine or control groups to receive three intramuscular immunizations of 50 μL (25 μL/leg), four weeks apart. Six groups of immunized mice (n = 40/group) were administered the SA antigen either non-adjuvanted or formulated with one of the five adjuvants. A saline control group (n = 40) received PBS only, and an untreated group (n = 3) was included as pre-immune controls for B-cell analyses. Pre- and post-treatment, mice were bled for serology or euthanized (five mice/group/timepoint) to harvest inguinal LNs (the proximal LNs draining the leg injections), spleens, and the BM for B-cell evaluations. Spleens were mechanically disrupted into cell suspensions, followed by a wash with Hanks’ balanced salt solution. After erythrocyte lysis, the splenic cells were washed, resuspended in PBS, and filtered. LNs were incubated with an enzymatic solution of Liberase (500 ug/mL; Sigma-Aldrich) and DNase (250 ug/mL; Thermo Fisher Scientific) for 1 h at 37°C, filtered, and centrifuged; then the cells were resuspended in PBS. Bone marrow washes collected from femurs were treated with erythrocyte lysis buffer; then the cells were washed and resuspended in PBS. Schedules of blood and tissue sample collection are presented in **Figure 1** and **Supplementary Figure S3**. In the similarly designed preliminary study, a total of 196 5-week-old female BALB/c mice received two injections of either the SA antigen formulated with one of the five adjuvants or without adjuvant, or of PBS only (n = 28/group). An untreated group (n = 3) was included as pre-immune controls for B-cell analyses (see **Supplementary Methods** for details). In both studies, animals were checked twice daily by qualified personnel, overseen by a veterinarian, as per the Veterinary Services’ internal procedures. The clinical status of all the animals was monitored before and during both studies, and signs of ill health or behavioral changes were recorded. No treatment-related clinical presentations were observed in any of the animals.

### 2.4 Binding antibody responses

Sera, standard, and controls were diluted in PBS using a Hamilton Microlab STAR system. The dilutions for Hla/CP5/CP8 were 1,000/1,000/1,000 (all vaccines) at pre-vaccination and 2 weeks post-dose 1, and 60,000/10,000/20,000 (non-adjuvanted group), 160,000/20,000/20,000 (Alum), 160,000/60,000/160,000 (AS01, AS04 and AS37) and 160,000/160,000/160,000 (AS03) at all timepoints thereafter. Diluted samples were added to 96-well plates (50 μL/well), and a mix of MagPlex Microspheres beads (12.5 × 10^6^/mL; DiaSorin, Saluggia, Italy) coupled to Hla, and avidin beads (Radix BioSolutions, Georgetown, TX, USA) coupled to CP5 and CP8, was added at 2,000 beads/antigen and mixed under agitation (60 min, 650 rpm). After two washes with PBS, R-Phycoerythrin AffiniPure F(ab’)_2_ fragment goat anti-mouse IgG secondary antibody (Jackson Immune Research, Ely, UK) was added to the wells (1:100) followed by incubation (15 min, 650 rpm). After three washes in PBS, the plates were read with a Luminex LX200 Instrument (Luminex Corp., Austin, TX, USA). Data were analyzed using Bio-Plex Manager software v6.1.1. (Bio-Rad, Hercules, CA, USA). The fluorescence intensity (FI) data for each dilution were interpolated into the standard curve and converted in RLU/mL, whereby the mean FI (MFI) at the first standard serum dilution (1:2,500) was considered to be 100 RLU/mL. For the saline group, a dilution of 1,000 was selected for all antigens and timepoints.

### 2.5 Antibody avidity

The Avidity Index (AI), i.e., the percentage of high-avidity antibodies within the total amount of antigen-specific antibodies, was determined using an avidity ELISA applied to a Gyrolab system with a 4-step method (‘capture,’ ‘sample,’ ‘PBS,’ ‘thiocyanate,’ ‘detection’) as previously described (29). FI data were analyzed using the Gyrolab workstation Control software and Gyrolab Evaluator software, and reaction profiles were visualized using the Gyrolab Viewer. AI values were calculated as follows: (FI measured after a wash with ammonium thiocyanate 1.5 M [Sigma-Aldrich] divided by the FI after a PBS wash) × 100. Samples were tested at dilutions of 1:300 (CP5, Hla), 1:1,000 (CP8), or, for the week 2 sera only, at 1:30 (all antigens) in Rexxip H buffer (Gyros Protein Technologies AB).

Biotinylated antigens (CP5-biot # MT 17/7/17 [1,028 μg/ml], CP8-biot # MT 17/7/17 [1,094 μg/ml] and Hla-H35L biot # 28/06/18 [655 μg/ml]) were used as capture reagents, each at 100 µg/mL (diluted in PBS/Tween20 0.01%). The Hla antigen was biotinylated using EZ-Link Sulfo-NHS-LC-Biotin (Thermo Fisher Scientific) at a molar excess of 10 mol of biotin:1 mol of protein. CP antigens were covalently bound to the biotin hydrazide by EDC activation of the carboxylic groups present on the D-mannosamine uronic acid (D-ManNAcA) in the repeating unit, in 100 mM MES buffer (pH 5). The detection reagent Alexa Fluor 647 AffiniPure F(ab’)2 fragment goat anti-mouse IgG (Jackson ImmunoResearch, Ely, UK; #115-606-071) was used at 25 nM. Dilutions were performed in Rexxip F buffer (Gyros Protein Technologies AB).

### 2.6 B-cell responses

Immunophenotyping and evaluation of the frequencies of antigen-specific MBCs and GC cells was performed using multiparametric flow cytometry; see **Supplementary Figure S1** for gating strategies. Total antigen-specific B cells were detected by labeling Hla with Alexa488, and biotinylating CP5 and CP8 (see above). Per mouse, 5 × 10^6^ lymphocytes from LNs and spleen were stained with Live/Dead NearIr (Invitrogen) for 20 min at room temperature [RT]), incubated (10 min, RT) with anti-CD16/CD32 Fc block (BD) and incubated with 0.1 μg Hla35L-Alexa488 or 0.6 μg CP5/CP8-biotine, with CD19-PE, IgM-BV421, IgD-Alexa700, CD38-PECy5, GL7-AlexaFluor647 for Hla detection, or GL7-FITC for CP detection, with CD80-PE-CF594, CD138-BV605, CD73-PECy7, and PD-L1-BV711 (1 h, 4°C) and washed with 1% FBS in PBS. The CP5/8-stained samples were then incubated with streptavidine-Alexa647 (20 min, 4°C) and washed with 1% FBS. Samples were fixed in BD Perm Buffer (15 min, on ice), resuspended in 2.5 mM PBS/EDTA, and acquired on an LSRII flow cytometer (BD Biosciences). Data were analyzed using FlowJo software v10 (TreeStar). For BM B cells, BM samples from five mice/group were pooled and stained with Live/Dead IR-Near (20 min, RT), incubated with anti-CD16/CD32 Fc-block (10 min, RT) and stained (1 h, 4°C) with the respective antigen as described above, but using CD19-PE, IgM-BV421, IgD-Alexa700, GL7-AlexaFluor647 (for Hla) or GL7-FITC (for CP5/8), and CD138-BV605 and B220-BV786.

### 2.7 Statistical analyses

The IgG response was analyzed using a linear mixed model applied to log10-transformed titers, implemented with the *lmer* package in R. This model included ‘treatment’ and ‘timepoint’ as fixed effects, and ‘animal ID’ as a random effect. To assess differences among groups and timepoints, pairwise comparisons were performed using the *pairs()* function from the *emmeans* package in R. The multivariate t (MVT) method was employed to adjust p-values and account for multiple testing. Results were reported as geometric mean titers (GMTs) and two-sided 95% confidence intervals (CIs), computed by base-10 exponentiation of the least squares (LS) means and the 95% CIs of the log_10_-transformed titers. The ratios of the GMTs (geometric mean ratios or GMRs) between two groups, along with their two-sided 95% CIs, were calculated by exponentiating the LS means and their 95% CIs obtained from the ANOVA model applied to the log_10_-transformed antibody titers.

Adjuvant effects on AI levels were assessed using generalized linear mixed-effects models with a β-distribution family, implemented through the *glmmTMB* package *in* R, with ‘treatment’ and ‘timepoint’ as fixed effects and ‘animal ID’ as a random effect. The β-dispersion parameter was fixed for Hla and allowed to vary for CP8 and CP5, as determined by comparing the models using a likelihood-ratio test. Intergroup differences were assessed using the *pairs()* function from the *emmeans* package in R, applied to back-transformed estimates. This approach allowed for direct comparison of the EMMs across different groups while accounting for the non-linear model applied. These comparisons were performed using the MVT method to adjust p-values for multiple comparisons, ensuring robust control of the type I error rate.

Splenic and LN B cell data were analyzed by antigen using generalized linear models with a negative binomial distribution, applied to the counts of MBC and GC cells. The model used was: *N (number of B cells) = group + timepoint + group ∗ timepoint*, with ‘group’ and ‘timepoint’ as fixed effects. Results were expressed as EMMs with 95% CI. Pairwise comparisons of EMMs derived from the generalized linear model were performed using the *pairs()* function in the *emmeans* package, applying the MVT method to adjust p-values for multiple testing. P values less than 0.05 were considered statistically significant. BM data were presented as pooled samples from five mice per group. All analyses were performed using Rstudio version 1.1.383.

## 3 Results

### 3.1 Greater adjuvant-mediated enhancement of IgG titers and persistence for CPs vs Hla

**Figure 2:**
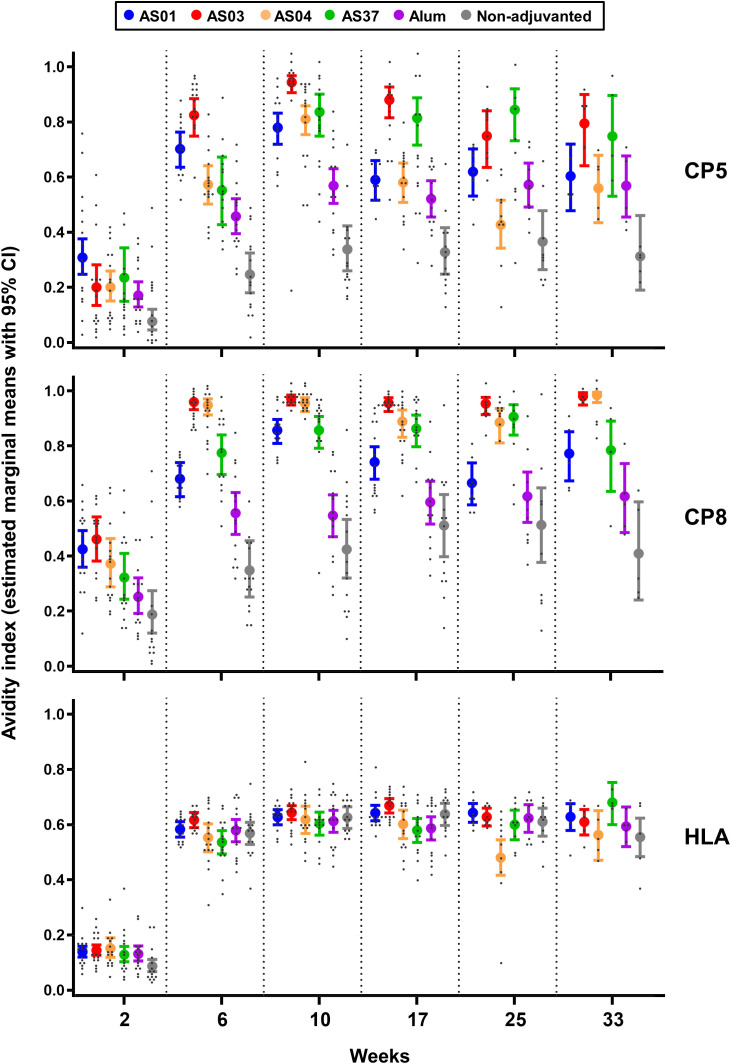
AS03 enhances avidity maturation of anti-polysaccharide IgG responses. Avidity indices (avidity titers over IgG titers) of antibodies specific for CP5, CP8 or Hla were measured 2 weeks after each immunization (weeks 2, 6, and 10) in sera from 16 mice/group, and at 2, 4, and 6 months post-dose 3 (weeks 17, 25, and 33) in sera from 15, 10, and 5 mice/group respectively (see **Figure 1A** for study design). Data were analyzed by fitting a mixed effect model to the data (with ‘group’ and ‘timepoint’ as fixed effects and their interaction included). Results are presented as estimated marginal means with 95% confidence intervals (closed circles with vertical bars, color-coded by group as shown in the key) and as individual data points (grey dots). Data from the saline-treated control group are not shown as the values were all near-zero.

**Figure 3:**
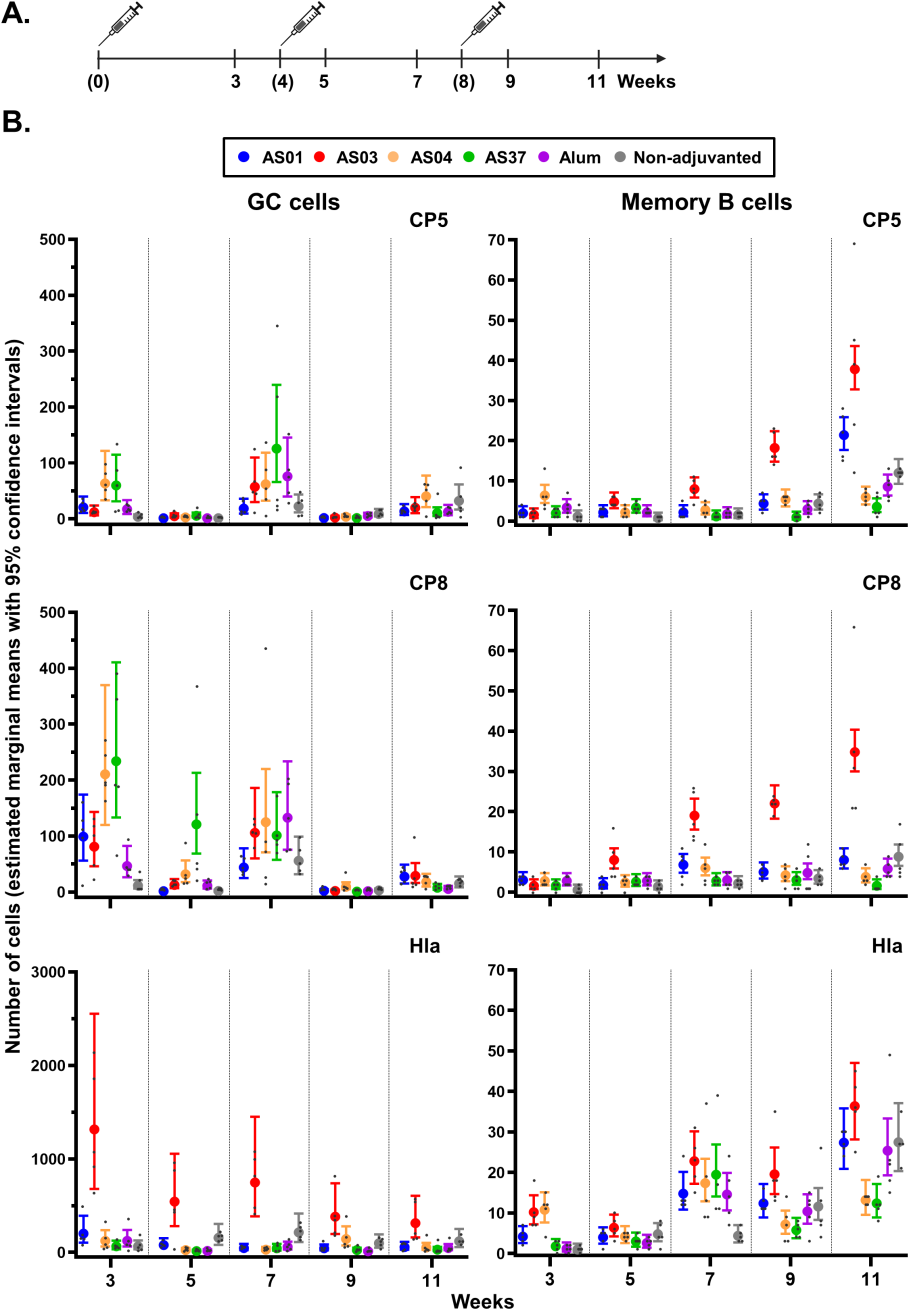
Antigen-specific B-cell expansion in the inguinal lymph nodes. **(A)** Schedule shows the timepoints of immunization (bracketed) and tissue collection for the proximal (inguinal) lymph nodes draining the immunization sites. Mice (N = 5/group) received three intramuscular injections with *Staphylococcus aureus* CP5-TT/CP8-TT/Hla vaccines that were either adjuvanted (with AS01, AS03, AS04, AS37, or Alum) or non-adjuvanted. A control group received saline only (data not shown as the values were all near-zero). **(B)** Numbers of B cells/million singlets of germinal center (GC) cells and antigen-specific memory B cells are presented by vaccine antigen. Data were analyzed by fitting a generalized linear model to the data with negative binomial assumption (with ‘group’ and ‘timepoint’ as fixed effects and their interaction included). Results are presented as estimated marginal means with 95% confidence intervals (closed circles with vertical bars, color-coded by group as shown in the key) and as individual data points (gray dots).

In the preparatory experiments (**Supplementary Methods**), a dose-dependent increase in mean titers (GMTs) was observed for each adjuvant (data not shown), and the highest doses tested (see *Materials and methods*) were selected for further evaluation. In the main study, mice received three immunizations, spaced 1 month apart, with either adjuvanted or non-adjuvanted formulations of the model antigen, or saline only, and were monitored for 25 weeks following the final immunization (**Figure 1A**). Statistical results of pairwise group and timepoint comparisons for **Figures 1**-**3**, with p-values adjusted for multiple testing, are provided in **Supplementary Data Files 1–4**.

After a single immunization, each AS-adjuvanted vaccine significantly increased CP5/8-specific antibody titers compared to the non-adjuvanted formulation (p <0.001; **Figure 1B**; **Supplementary Data File 1**). The second immunization significantly boosted these primary titers in all vaccine groups (p <0.001), suggesting the presence of CP5/8-specific MBCs. Two weeks after a second or third dose, CP5/8-specific GMTs in the AS-adjuvanted groups exceeded those in the Alum (p <0.001), consistent with previously reported effects of AS37 and AS04 on murine anti-polysaccharide responses to other antigens (4, 19). A modest increase in CP5-specific titers (but not CP8-specific titers) was observed after the third dose in all vaccine groups (p ≤0.04). CP5/8-specific titers plateaued by week 10 or 17 and persisted through at least 25 weeks after the third dose.

As expected (30), the Hla protein was highly immunogenic when administered alone. Although Hla-specific titers for each adjuvanted vaccine exceeded those of the non-adjuvanted vaccine after the second or third dose (p <0.001), none of the adjuvants had a statistically significant effect on long-term persisting Hla-specific antibody titers at 25 weeks after the third dose.

Overall, the titers elicited by the AS-adjuvanted vaccines were comparable across the four groups for both the proteic and polysaccharidic antigens. This was expected, as the AS doses were selected based on preparatory experiments to minimize the variability between groups, thereby allowing focus on the qualitative features of the immune response.

The adjuvant effects were corroborated by analyzing the geometric mean ratios (GMRs) of the titers in each adjuvanted group relative to the non-adjuvanted group at 2 weeks or ≥9 weeks after each immunization (**Figure 1C**; upper or lower panels, respectively). In particular, following the second immunization with AS03, the increase in IgG response relative to the non-adjuvanted group was robust for the polysaccharidic antigens but modest for the protein antigen, and this pattern persisted at 25 weeks post-dose 3 (GMRs for CP5, CP8 and Hla: 83, 130, and 4 [week 6], and 16, 22 and 1 [week 33], respectively). Overall, these results highlight a clear difference in the adjuvants’ effects on the antibody response to polysaccharide versus protein antigens, with greater enhancements in both the magnitude and persistence of the IgG response to CP5/8 compared to Hla.

### 3.2 AS03 mediates the highest levels of avidity maturation for anti-CP antibodies

Having established the distinct adjuvant effects for the two antigen types, we next asked whether these differences were reflected in antibody avidity. Proportions of high-avidity antibodies within the total IgG response, as represented by the EMMs of the avidity index (AI), were determined using a Gyros-based assay. Highly overlapping results from three independent experiments suggested that the assay was robust (**Supplementary Figure S2A**).

The EMMs of high-avidity CP5/8-specific antibodies increased after each immunization, suggesting the presence of avidity maturation (**Figure 2**, **Supplementary Data File 2**). Values in the AS groups exceeded those in the non-adjuvanted and Alum groups. Among the AS groups, CP5-specific EMMs for AS03 were higher than those for AS01 and AS04 at 2 weeks post-dose 3 (0.94 versus 0.78 and 0.81, respectively; p <0.001), but had decreased 23 weeks later. At this persistence time point, EMMs were not statistically different among the AS groups (range: 0.79–0.56). CP8-specific EMMs for AS03 and AS04 remained relatively constant from 2 weeks post-dose 2 onward (range: 0.89–0.99) and exceeded levels for the other AS formulations at both 2 and 25 weeks post-dose 3 (p ≤0.03).

Remarkably, the EMMs of high-avidity anti-Hla antibodies increased significantly after the second immunization (p <0.001) to levels that were similar across all formulations (range: 0.54–0.64), and remained stable at subsequent time points.

Comparable trends among the adjuvants were observed for the avidity of CP8-specific antibodies measured after two immunizations in a smaller study using the same model antigen (**Supplementary Figure S2B**).

Since high-avidity antibodies are secreted by plasma cells derived from mature MBCs that have differentiated through the GC reaction, these findings suggest that AS03 mediated the strongest affinity maturation of CP-specific B cell responses in the GCs.

### 3.3 Improved quality of anti-CP antibody responses can be linked to the local B cell response

In GC reactions, which occur primarily in the draining lymph nodes (dLNs) and spleen (31), recruited B cell clones proliferate and undergo somatic hypermutation. Upon antigen recall, MBCs are selected for further affinity maturation or for differentiation into higher-affinity, antibody-secreting plasma cells (4, 32). To investigate whether the avidity maturation of the anti-CP antibodies was linked to the local MBC and GC B-cell response, antigen-specific isotype-switched (CD19^+^ IgD^−^ IgM^−^) GL7^+^ GC B cells and mature (GL7^−^CD38^+^CD73^+^ CD80^+^) MBCs in the dLNs were quantified after each dose by flow cytometry (see **Figure 3A** [study design], **Supplementary Figure S1** [gating strategies], and **Supplementary Data File 3** [statistics]).

For all adjuvants, CP5/8-specific GC B cell counts were slightly increased (means <234) three weeks after the first immunization, and for CP8 also 1 week after the second immunization. All levels had returned to baseline by 1 or 3 weeks post-dose 3, aligning with the expected lifetime of vaccine-induced GCs of approximately 3 weeks (33, 34) (**Figure 3B**). Only AS03 induced a significant expansion of the MBC population after the first antigen recall (week 7 versus week 3: p <0.001), which increased further post-dose 3 (week 11 vs. week 7: p <0.001; fold-change: 4.7 and 1.9 for CP5 and CP8, respectively).

Different patterns were observed for the Hla-specific responses: GC B cells counts increased only with AS03, and primarily after the first immunization (EMM at week 3: 1,317), whereas MBC counts increased across formulations and after each immunization (EMMs ≤36). Thus, the AS03-mediated effects observed in the antibody avidity maturation may be linked to the impact of this adjuvant on the local polysaccharide-specific B cell response.

### 3.4 AS03 stimulates the systemic polysaccharide-specific B cell response

**Figure 4:**
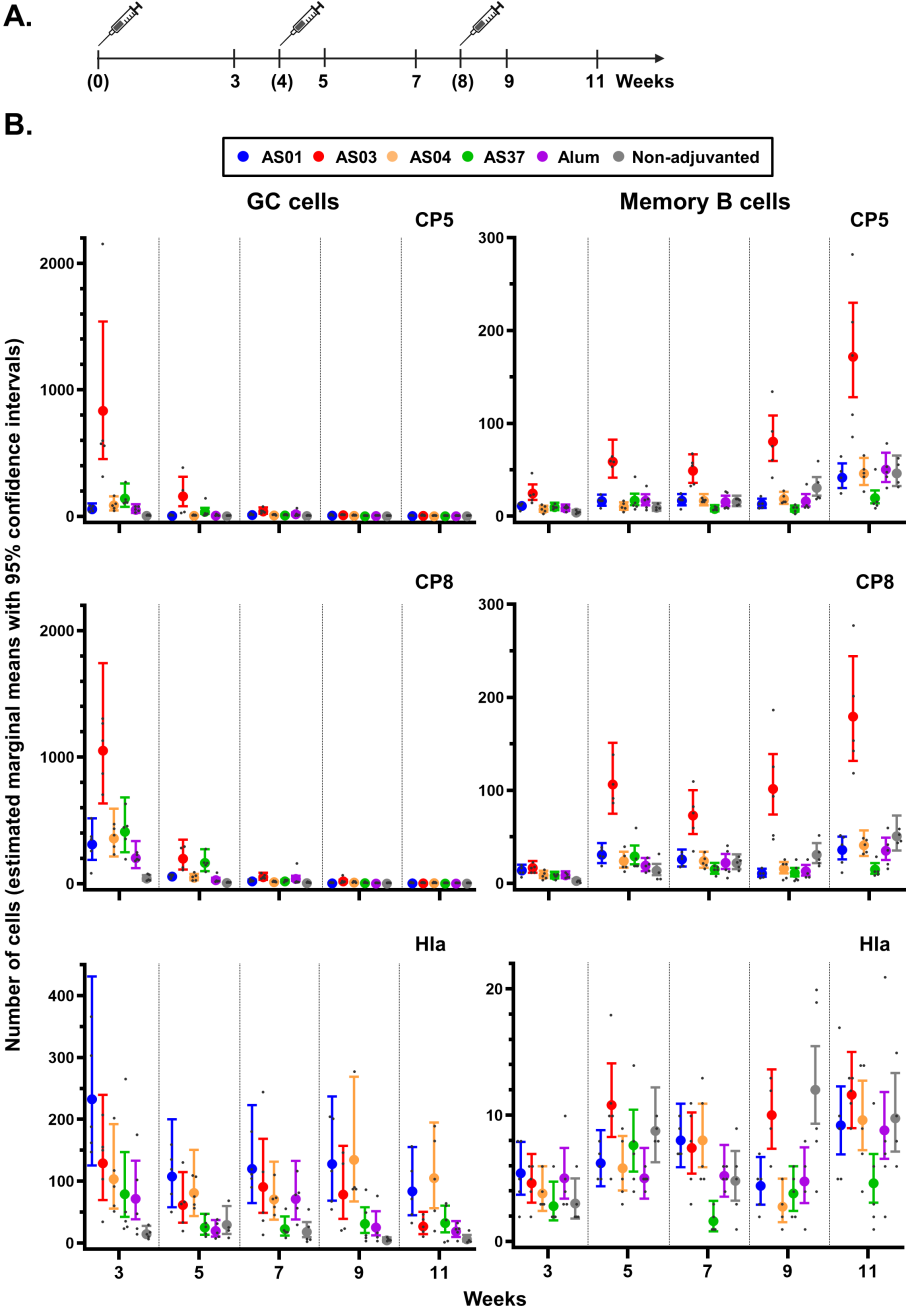
Antigen-specific B-cell expansion in the spleen. **(A)** Schedule shows the timepoints of immunization (bracketed) and tissue collection for the spleen. Mice (N = 5/group) received three intramuscular injections with *Staphylococcus aureus* CP5-TT/CP8-TT/Hla vaccines that were either adjuvanted (with AS01, AS03, AS04, AS37, or Alum) or non-adjuvanted. A control group received saline only (data not shown as the values were all near-zero). **(B)** Numbers of B cells/million singlets of antigen-specific germinal center (GC) cells and antigen-specific memory B cells are presented by vaccine antigen. Data were analyzed by fitting a generalized linear model to the data with negative binomial assumption (with ‘group’ and ‘timepoint’ as fixed effects and their interaction included). Results are presented as estimated marginal means with 95% confidence intervals around the estimated marginal mean (closed circles with vertical bars, color-coded by group as shown in the key) and as individual data points (gray dots).

As most long-lived MBCs reside in the spleen, we next compared adjuvant effects on local B cell responses with those in the spleen, using the same experimental setup as for the dLN (see **Figure 4A** for study design and **Supplementary Figure S1** for gating strategy). Statistics for pairwise group and timepoint comparisons are provided in **Supplementary Data File 4**.

A CP5/8-specific GC reaction was primarily observed after the first dose (**Figure 4B**). Adjuvant effects on CP5/8-specific GC B cell responses were detected in all adjuvant groups (p <0.001 versus non-adjuvanted vaccine). Notably, AS03 induced higher CP5-specific responses compared to the other AS formulations (p <0.001), and higher CP8-specific responses as compared to AS01 and AS04 (p ≤0.04). Only AS03 elicited a significant expansion of CP5/8-specific MBC beginning 1 week post-dose 2 onwards (p <0.001 vs. the other adjuvants). This expansion continued through 3 weeks post-dose 3 (e.g., an 11-fold change at week 11 over week 3 for CP8).

The Hla-specific B-cell responses exhibited distinct kinetics, with all adjuvants inducing a GC response starting from the first dose. Although some TLR-based adjuvants are known to prolong GC responses to protein antigens (35, 36), no clear differences among AS01, AS04, and the emulsion-based adjuvant AS03 were observed up to week 9. Similarly, no consistent differences among the AS were observed in splenic Hla-specific MBC responses, reflecting the patterns seen in the dLN MBCs (see **Figure 3**).

Because splenic mature MBCs largely mediate secondary B-cell responses at distal sites, we evaluated the effects of adjuvants on the induction of LLPCs persisting in the bone marrow up to 25 weeks post-final immunization (**Supplementary Figure S3**; data from one pool of five mice per group). Although statistical analyses were not performed, expansions of polysaccharide-specific LLPC populations at 9 weeks post-dose 3 were greater for AS03 compared to the other adjuvants (CP5/8: 600/300 cells vs. <150/<150 cells). These responses returned to baseline–8 or 16– weeks later in all groups. No intergroup differences were observed in the Hla-specific responses, which remained low (<100 cells) throughout all study timepoints.

Overall, the differences in antibody titers between Hla-specific and CP-specific responses were reflected by both local and systemic MBC responses. Thus, by promoting GC reactions in local and systemic sites, AS03 as the most effective adjuvant for enhancing and sustaining the responses of mature MBCs and LLPCs, two key components of the humoral memory compartment.

## 4 Discussion

Vaccines that contain protein-polysaccharide conjugates, or glycoconjugates, are vital tools to address the emergence of AMR. Although numerous studies have shown that combination adjuvants like the AS families enhance B-cell responses to protein antigens (6–9, 23–27), their effects and mechanisms of action in the context of glycoconjugates are still not well understood (37, 38). In this study, we evaluated immune responses in naïve to model glycoconjugate antigens (CP5-TT/CP8-TT) and a protein antigen (Hla). We found that vaccines formulated with AS01, AS03, AS04 or AS37 significantly increased CP5 and CP8-specific antibody titers and avidity compared to Alum- or non-adjuvanted formulations. Among the adjuvants tested, AS03 elicited the most pronounced CP-specific immune responses, characterized by robust high-avidity antibodies. In the dLN, all adjuvants promoted expansion of CP5- and/or CP8-specific GC B cells, but only AS03 led to a significantly larger MBC compartment. A similar pattern was observed systenmically: AS03 induced an early and sustained expansion of the CP-specific GC B-cell population, which corresponded to a markedly stronger increase in CP-specific MBCs over time. Consistently, LLPC responses to CP antigens in the bone marrow were predominantly induced by AS03. The effects of AS03 on CP-specific immunity grew stronger with each immunization, suggesting ongoing B-cell maturation and increased antibody affinity, and persisted for at least 25 weeks after the final dose. These findings in a naïve model underscore the role of a carrier protein in enabling AS03 to enhance glycoconjugate-specific immunity by improving the quality of the GC response. In contrast, Hla-specific responses showed similar GC and MBC dynamics across all adjuvants, except for a strong AS03-induced increase in GC B cells in the dLN, which did not result in a significantly greater MBC expansion. Overall, our findings highlight that evaluating both antibody titers and avidity, alongside local and systemic B-cell population dynamics, is essential for understanding how different adjuvants shape immune responses to protein–polysaccharide antigens.

The enhancements in anti-polysaccharide antibody titers and avidity observed with the four AS adjuvants, relative to Alum, are consistent with murine data from glycoconjugate vaccines adjuvanted with AS04, AS37, or o/w emulsions, as well as with dLN MBC responses elicited by an AS37-adjuvanted model vaccine (4, 18–20). Although the strong immunogenicity of Alum-formulated vaccines in human participants may have obscured some differences, our findings align with the trend toward higher titers seen with AS37- vs. Alum-adjuvanted MenC-CRM_197_ vaccines in young adults (11, 14), emphasizing the importance of conducting mechanistic studies in naïve models. Notably, in this trial, the AS37-adjuvanted MenC vaccine also induced Tfh (14) cell responses, which are key regulators of the initiation, quality, magnitude, and durability of the GC B cell response (39). Overall, our findings extend existing data on protein-based viral antigens in both animal models and humans by demonstrating that AS adjuvants elicit immune responses that are both quantitatively and qualitatively distinct from those induced by Alum, supporting our initial hypothesis (6–9, 24, 25, 27, 40–43).

AS03 appeared to be most effective in enhancing the avidity maturation of anti-CP antibodies. As dissected for protein antigens in animal models, this adjuvant works at the injection site by activating an endoplasmic reticulum stress sensor (IRE1α) in myeloid cells, stimulating pro-inflammatory cytokine and APC responses (44, 45). In the dLN, this was shown to promote Th and Tfh cell responses, which interact with APCs to initiate the B cell response in the GCs, resulting in high-affinity antibodies. Similarly, AS03 was shown in humans to activate naïve B cells, improve memory recall, and induce peripheral antigen-specific CD4^+^ T cell responses and increased Tfh responses (6, 46–49). In fact, CD4^+^ T-cell responses against the TT carrier were also observed in primed adults who received the AS03-adjuvanted four-component SA glycoconjugate vaccine (22). Our data suggest that AS03 provided a stronger stimulation of Th-cell help and B-cell improvement as compared to the other adjuvants, and this initial adjuvant effect created, after the second and third dose, mature MBC populations, which could be linked to a markedly improved splenic GC reaction. In contrast, little interaction between these two responses could be detected for the other adjuvants or non-adjuvanted group. Further research, focusing on Tfh cell responses to these adjuvants in the dLN, could test these hypotheses. Finally, the data also suggest that at least two doses of AS03-adjuvanted CP antigens are required for the induction of persistent high-avidity antibody responses in these naive animals. This may hold particularly true for the CP5-specific antibody responses and may be aligned with the antibody responses reported for an MF59-adjuvanted glycoconjugate vaccine in naïve non-human primates (20).

The increased avidity was aligned with the adjuvant’s ability to drive qualitative improvements in the antibody response. Indeed, in humans, AS03 was shown to increase antibody avidity and other functional antibody characteristics, such as Fc-mediated features, compared with Alum or AS04 (42, 43). Increased functionality was also demonstrated for the four-component SA glycoconjugate vaccine (22), showing that AS03 enhanced the responses of antibodies blocking clumping factor A (one of the vaccine antigens) binding to fibrinogen, and did so already after a single dose. This could be relevant for rapid response vaccination strategies to counter nosocomial infections (38). Furthermore, the observed longevity of the CP5/8-specific MBC response of at least 25 weeks post-immunization, along with the trend for higher responses of bone marrow LLPCs, suggests that AS03 also enhances the persistence of anamnestic B cell responses compared with Alum or no adjuvant. Similar observations have been made for AS03-adjuvanted protein antigens in non-human primates and humans (42, 47, 50, 51). Altogether, the trends observed here converge with those found previously in animal models or humans for protein-based or glycoconjugate vaccines, suggesting that our SA study vaccine is a suitable model vaccine for our research objectives.

Our data also suggest that the current observations could be sensitive to the immune memory status and that in the presence of preexisting immunity, the benefit of AS03 may lie more in qualitative than in quantitative improvements of the antibody response and MBC pool. This feature may be interesting for vaccine development targeting older and immunocompromised populations who have reduced abilities to form naive B cells, new GCs, and GC B-cell responses. Indeed, in these individuals, the adjuvant could help to potentiate the production of high-affinity LLPCs and MBCs and overcome the effects of GC exhaustion (15). Overall, our data confirm and extend reports for protein-based vaccines that identified AS03 as an effective agent to improve the B-cell response. As these features allowed antigen sparing, cross-clade protection, and large-scale vaccine manufacturing, AS03 has been incorporated in protein-based (pre)pandemic vaccines against H5N1 and H1N1 influenza strains and, more recently, in a European Medicines Agency (EMA)-authorized SARS-CoV-2 booster vaccine (12, 52–54). To further define the mechanism for glycoconjugate vaccines, a research avenue might be into any AS03-mediated activation of pathways underpinning the regulation of B-cell activation and affinity maturation in LLPCs, such as oxidative phosphorylation (55, 56).

Aligned with human data on the effects of AS02 (oil-in-water emulsion combining MPL and QS-21) and Alum to conjugated pneumococcal vaccine (57), our post-dose 2 data pointed to distinct adjuvant effects on humoral and B-cell responses between the protein and the polysaccharide antigens tested. Indeed, compared to the non-adjuvanted vaccine, effects on titers after the third dose were more pronounced for the CPs than for Hla, likely due to the high immunogenicity of the latter (30). Furthermore, avidity maturation and inter-group differences in the avidity data were clearly apparent in the anti-CP responses, but not in the anti-Hla responses after the second or third dose. Similarly, while the CP-specific B-cell responses were mostly clearly different between the adjuvants, no large intergroup differences were observed in the Hla-specific responses of dLN/splenic MBCs, splenic GC cells, or bone marrow LLPCs. The exception was the higher GC reaction in the dLN for AS03 for Hla-specific responses, which did not translate into a significantly larger expansion of the dLN MBC population, higher Hla-specific titers, or higher levels of avidity maturation for AS03 vs the other adjuvants. This needs further study. Furthermore, the Hla data obtained in the current mouse model did not display the previously reported adjuvant ranking for antibody titers/avidity and B-cell responses to HBsAg (AS01_E_/AS03 > AS04 ≥ Alum) seen in humans who were HBsAg-naive at pre-vaccination (42). This may be explained by the differences in the immunogenicity of these two protein antigens, as seen previously in mice. Indeed, for a protein-based SA vaccine, Alum- and an o/w emulsion-adjuvanted vaccines were equally immunogenic with respect to Hla-specific IgG responses (58), while responses to HBsAg displayed clear differences between the AS03-, Alum-, and non-adjuvanted vaccines (45). A deeper understanding of how the interactions between adjuvants and physicochemical features of the antigen could affect such immune system processes is therefore needed.

The study limitations include the small size of the bone marrow cell samples used in the long-term memory assessment. Sufficient robustness of our other results was obtained by performing both a preliminary and a main study, the latter generating single data points to appreciate the inter-individual variability in the data. Nonetheless, as the impact of these adjuvants on dLN-resident Tfh cell populations was not assessed, the link between such T-cell responses and the observed differences in antibody avidity between the adjuvants remains to be elucidated. Moreover, due to interspecies differences in the signaling pathways and expression of some TLRs receptors (59), the reproducibility of the adjuvant-specific data in humans, particularly for primed individuals in a real-world setting, remains unclear. Finally, the translatability of our vaccine immunogenicity data to protection against challenge SA infection or to immune responses elicited by differently manufactured constructs such as bioconjugates [*in vivo* conjugation of polysaccharides to proteins (3)] remains to be investigated.

In conclusion, our data provide a proof of concept that in a naive setting, the immunogenicity of glycoconjugate vaccines, including the tested antigens, can benefit from the use of adjuvants. The data also suggest that AS03 may represent a promising adjuvant to promote increased functional memory formation by these vaccines by affecting both the polysaccharide- and protein-specific antibody and B-cell responses. Investigating both the quality of the humoral response and the antigen-specific B-cell expansion helps to understand the role of adjuvants in shaping the response to glycoconjugate vaccines, which could ultimately facilitate the development of antibacterial vaccines.

## Author’s note

AS01, AS03, and Engerix-B are trademarks owned by or licensed to the GSK group of companies.

## Data Availability

The original contributions presented in the study are included in the article/**Supplementary Material**. Further inquiries can be directed to the corresponding author.

## Supplementary material

Supplementary Figure 1 B-cell gating strategies. Flow cytometry gating strategies for phenotypic characterization of antigen-specific B cells in the lymph nodes or spleens **(A)** or bone marrow **(B)** are presented. Gating for the antigen-specific populations for immunized mice was selected based on the background derived from phosphate-buffered saline (PBS)-injected mice. **(A)** Cells were selected based on viability, morphology, and singlets, and B cells were identified as CD19^+^ cells. Switched (IgD^-^ IgM^-^) B cells were analyzed for CP5/8 (CP) or Hla positivity. CP^+^ and Hla^+^ B cells were further defined by GL7 expression (to identify cells involved in germinal center [GC] reactions) and CD38 expression, which, combined with GL7 downregulation, identified mature B cells about to exit the lymphoid organ. CD38^+^ GL7^-^ cells were analyzed for CD80 and CD73 expression to identify the mature memory B cells (MBC) that underwent somatic mutation after encountering the antigen. **(B)** Bone marrow cells were selected based on CD19 expression, viability, morphology, and singlets. Switched (IgD^-^ IgM^-^) B cells were analyzed for expression of B220 (downregulated in B cells differentiated into plasma cells [PC]) and GL7, to identify B cells involved in GC reactions. B220^-^ GL7^-^ cells were analyzed for CD138 expression to detect PC, and the CD138^+^ subset was analyzed for antigen positivity.

Supplementary Figure 2 Antibody avidity preliminary study. Mice (n=8/group) received two intramuscular injections, four weeks apart, of a CP5-TT/CP8-TT/Hla vaccine that was either adjuvanted (with AS01, AS03, AS04, AS37, or Alum), or non-adjuvanted. Blood samples were collected two weeks after the second dose. **(A)** The avidity indices of antigen-specific antibodies of each individual serum sample were measured in three separate experiments. Each symbol represents a single measurement for one individual animal. **(B)**. Estimated marginal means (EMMs; colored dots) of the avidity indices derived from a mixed effect model are presented with 95% confidence intervals (CI; grey vertical bars).

Supplementary Figure 3 Antigen-specific B-cell expansion in the bone marrow. **(A)** Schedule shows the timepoints of the treatments with *Staphylococcus aureus* vaccines containing non-adjuvanted and adjuvanted CP5, CP8, and Hla antigens (bracketed) and tissue collection timepoints of the bone marrow samples at 9, 17, and 25 weeks post-dose 3 (pIII). Mice received three intramuscular injections of CP5-TT/CP8-TT/Hla vaccine either adjuvanted with Alum, AS01, AS03, AS04 or AS37, or non-adjuvanted. A control group received phosphate-buffered saline (PBS). For each group, samples from 5 mice were combined into a single pool. **(B)** Numbers of antigen-specific long lived plasma cells/million singlets are presented as a single data point per group, color-coded as shown in the key.

Supplementary Table 1 Design adjuvant dose-finding experiments. *A/C, active/control groups. **volume-based ratios for 25 μL AS03 + 25 μL antigen mixture. Bold font, dose that was ultimately selected for further experiments. Alum, aluminum hydroxide. AS37 contained a synthetic TLR7 agonist (TLR-7a) adsorbed on Alum.

## References

[1] Micoli F Bagnoli F Rappuoli R Serruto D. The role of vaccines in combatting antimicrobial resistance. Nat Rev Microbiol. (2021) 19:287–302. doi: 10.1038/s41579-020-00506-3, PMID: 33542518 PMC7861009

[2] Frost I Sati H Garcia-Vello P Hasso-Agopsowicz M Lienhardt C Gigante V. The role of bacterial vaccines in the fight against antimicrobial resistance: an analysis of the preclinical and clinical development pipeline. Lancet Microbe. (2023) 4:e113–25. doi: 10.1016/S2666-5247(22)00303-2, PMID: 36528040 PMC9892012

[3] Romano MR Berti F Rappuoli R. Classical- and bioconjugate vaccines: comparison of the structural properties and immunological response. Curr Opin Immunol. (2022) 78:102235. doi: 10.1016/j.coi.2022.102235, PMID: 35988326

[4] Rappuoli R. Glycoconjugate vaccines: Principles and mechanisms. Sci Transl Med. (2018) 10:eaat4615. doi: 10.1126/scitranslmed.aat4615, PMID: 30158151

[5] Sorieul C Dolce M Romano MR Codée J Adamo R. Glycoconjugate vaccines against antimicrobial resistant pathogens. Expert Rev Vaccines. (2023) 22:1055–78. doi: 10.1080/14760584.2023.2274955, PMID: 37902243

[6] Leroux-Roels G Marchant A Levy J Van Damme P Schwarz TF Horsmans Y. Impact of adjuvants on CD4^+^ T cell and B cell responses to a protein antigen vaccine: Results from a phase II, randomized, multicenter trial. Clin Immunol. (2016) 169:16–27. doi: 10.1016/j.clim.2016.05.007, PMID: 27236001

[7] Burny W Callegaro A Bechtold V Clement F Delhaye S Fissette L. Different adjuvants induce common innate pathways that are associated with enhanced adaptive responses against a model antigen in humans. Front Immunol. (2017) 8:943. doi: 10.3389/fimmu.2017.00943, PMID: 28855902 PMC5557780

[8] Burny W Marchant A Hervé C Callegaro A Caubet M Fissette L. Inflammatory parameters associated with systemic reactogenicity following vaccination with adjuvanted hepatitis B vaccines in humans. Vaccine. (2019) 37:2004–15. doi: 10.1016/j.vaccine.2019.02.015, PMID: 30850240

[9] De Mot L Bechtold V Bol V Callegaro A Coccia M Essaghir A. Transcriptional profiles of adjuvanted hepatitis B vaccines display variable interindividual homogeneity but a shared core signature. Sci Transl Med. (2020) 12:eaay8618. doi: 10.1126/scitranslmed.aay8618, PMID: 33177181

[10] Pulendran B Arunachalam S O’Hagan DT. Emerging concepts in the science of vaccine adjuvants. Nat Rev Drug Discov. (2021) 20:454–75. doi: 10.1038/s41573-021-00163-y, PMID: 33824489 PMC8023785

[11] Gonzalez-Lopez A Oostendorp J Koernicke T Fadini T D’Oro U Baker S. Adjuvant effect of TLR7 agonist adsorbed on aluminum hydroxide (AS37): A phase I randomized, dose escalation study of an AS37-adjuvanted meningococcal C conjugated vaccine. Clin Immunol. (2019) 209:108275. doi: 10.1016/j.clim.2019.108275, PMID: 31669193

[12] O’Hagan DT van der Most R Lodaya RN Coccia M Lofano G. World in motion” - emulsion adjuvants rising to meet the pandemic challenges. NPJ Vaccines. (2021) 6:158. doi: 10.1038/s41541-021-00418-0, PMID: 34934069 PMC8692316

[13] D’Oro U O’Hagan DT. The scientific journey of a novel adjuvant (AS37) from bench to bedside. NPJ Vaccines. (2024) 9:26. doi: 10.1038/s41541-024-00810-6, PMID: 38332005 PMC10853242

[14] Siena E Schiavetti F Borgogni E Taccone M Faenzi E Brazzoli M. Systems analysis of human responses to an aluminium hydroxide-adsorbed TLR7 agonist (AS37) adjuvanted vaccine reveals a dose-dependent and specific activation of the interferon-mediated antiviral response. Vaccine. (2023) 41:724–34. doi: 10.1016/j.vaccine.2022.12.006, PMID: 36564274

[15] Stefanetti G Borriello F Richichi B Zanoni I Lay L. Immunobiology of carbohydrates: implications for novel vaccine and adjuvant design against infectious diseases. Front Cell Infect Microbiol. (2021) 11:808005. doi: 10.3389/fcimb.2021.808005, PMID: 35118012 PMC8803737

[16] Taylor DN Trofa AC Sadoff J Chu C Bryla D Shiloach J. Synthesis, characterization, and clinical evaluation of conjugate vaccines composed of the O-specific polysaccharides of *Shigella dysenteriae* type 1, *Shigella flexneri* type 2a, and *Shigella sonnei* (*Plesiomonas shigelloides*) bound to bacterial toxoids. Infect Immun. (1993) 61:3678–87. doi: 10.1128/iai.61.9.3678-3687.1993, PMID: 8359890 PMC281064

[17] Wuorimaa T Dagan R Eskola J Janco J Ahman H Leroy O. Tolerability and immunogenicity of an eleven-valent pneumococcal conjugate vaccine in healthy toddlers. Pediatr Infect Dis J. (2001) 20:272–7. doi: 10.1097/00006454-200103000-00011, PMID: 11303829

[18] Vo HTM Baudner BC Sammicheli S Iannacone M D’Oro U Piccioli D. Alum/Toll-Like receptor 7 adjuvant enhances the expansion of memory B cell compartment within the draining lymph node. Front Immunol. (2018) 9:641. doi: 10.3389/fimmu.2018.00641, PMID: 29686670 PMC5900039

[19] Buonsanti C Balocchi C Harfouche C Corrente F Galli Stampino L Mancini F. Novel adjuvant Alum-TLR7 significantly potentiates immune response to glycoconjugate vaccines. Sci Rep. (2016) 6:29063. doi: 10.1038/srep29063, PMID: 27439378 PMC4954951

[20] Granoff DM McHugh YE Raff HV Mokatrin AS Van Nest GA. MF59 adjuvant enhances antibody responses of infant baboons immunized with *Haemophilus influenzae* type b and *Neisseria meningitidis* group C oligosaccharide-CRM_197_ conjugate vaccine. Infect Immun. (1997) 65:1710–5. doi: 10.1128/iai.65.5.1710-1715.1997, PMID: 9125551 PMC175202

[21] Fiorino F Rondini S Micoli F Lanzilao L Alfini R Mancini F. Immunogenicity of a bivalent adjuvanted glycoconjugate vaccine against *Salmonella Typhimurium* and Salmonella Enteritidis. Front Immunol. (2017) 8:168. doi: 10.3389/fimmu.2017.00168, PMID: 28289411 PMC5326758

[22] Levy J Licini L Haelterman E Moris P Lestrate P Damaso S. Safety and immunogenicity of an investigational 4-component *Staphylococcus aureus* vaccine with or without AS03_B_ adjuvant: results of a randomized phase I trial. Hum Vaccin Immunother. (2015) 11:620–31. doi: 10.1080/21645515.2015.1011021, PMID: 25715157 PMC4514337

[23] Fochesato M Dendouga N Boxus M. Comparative preclinical evaluation of AS01 versus other Adjuvant Systems in a candidate herpes zoster glycoprotein E subunit vaccine. Hum Vaccin Immunother. (2016) 12:2092–5. doi: 10.1080/21645515.2016.1154247, PMID: 26933767 PMC4994747

[24] Dendouga N Fochesato M Lockman L Mossman S Giannini SL. Cell-mediated immune responses to a varicella-zoster virus glycoprotein E vaccine using both a TLR agonist and QS21 in mice. Vaccine. (2012) 30:3126–35. doi: 10.1016/j.vaccine.2012.01.088, PMID: 22326899

[25] Liang F Lindgren G Sandgren KJ Thompson EA Francica JR Seubert A. Vaccine priming is restricted to draining lymph nodes and controlled by adjuvant-mediated antigen uptake. Sci Transl Med. (2017) 9:eaal2094. doi: 10.1126/scitranslmed.aal2094, PMID: 28592561

[26] Isaacs A Li Z Cheung STM Wijesundara DK McMillan CLD Modhiran N. Adjuvant selection for influenza and RSV prefusion subunit vaccines. Vaccines (Basel). (2021) 9:71. doi: 10.3390/vaccines9020071, PMID: 33498370 PMC7909420

[27] Bouzya B Rouxel RN Sacconnay L Mascolo R Nols L Quique S. Immunogenicity of an AS01-adjuvanted respiratory syncytial virus prefusion F (RSVPreF3) vaccine in animal models. NPJ Vaccines. (2023) 8:143. doi: 10.1038/s41541-023-00729-4, PMID: 37773185 PMC10541443

[28] Leuzzi R Bodini M Thomsen IP Soldaini E Bartolini E Muzzi A. Dissecting the human response to *Staphylococcus aureus* systemic infections. Front Immunol. (2021) 12:749432. doi: 10.3389/fimmu.2021.749432, PMID: 34819932 PMC8607524

[29] Piccioli D Buricchi F Bacconi M Bechi N Galli B Ferlicca F. Enhanced systemic humoral immune response induced in mice by Generalized Modules for Membrane Antigens (GMMA) is associated with affinity maturation and isotype switching. Vaccines (Basel). (2023) 11:1219. doi: 10.3390/vaccines11071219, PMID: 37515035 PMC10384117

[30] Karauzum H Updegrove TB Kong M Wu IL Datta SK Ramamurthi KS. Vaccine display on artificial bacterial spores enhances protective efficacy against *Staphylococcus aureus* infection. FEMS Microbiol Lett. (2018) 365:fny190. doi: 10.1093/femsle/fny190, PMID: 30084923 PMC6454432

[31] Shinnakasu R Inoue T Kometani K Moriyama S Adachi Y Nakayama M. Regulated selection of germinal-center cells into the memory B cell compartment. Nat Immunol. (2016) 17:861–9. doi: 10.1038/ni.3460, PMID: 27158841

[32] Cancro MP Tomayko MM. Memory B cells and plasma cells: The differentiative continuum of humoral immunity. Immunol Rev. (2021) 303:72–82. doi: 10.1111/imr.13016, PMID: 34396546

[33] Arulraj T Binder SC Meyer-Hermann M. Investigating the mechanism of germinal center shutdown. Front Immunol. (2022) 13:922318. doi: 10.3389/fimmu.2022.922318, PMID: 35911680 PMC9329532

[34] Pedersen GK Wørzner K Andersen P Christensen D. Vaccine adjuvants differentially affect kinetics of antibody and germinal center responses. Front Immunol. (2020) 11:579761. doi: 10.3389/fimmu.2020.579761, PMID: 33072125 PMC7538648

[35] Kasturi SP Skountzou I Albrecht RA Koutsonanos D Hua T Nakaya HI. Programming the magnitude and persistence of antibody responses with innate immunity. Nature. (2011) 470:543–7. doi: 10.1038/nature09737, PMID: 21350488 PMC3057367

[36] Rookhuizen DC DeFranco AL. Toll-like receptor 9 signaling acts on multiple elements of the germinal center to enhance antibody responses. Proc Natl Acad Sci U S A. (2014) 111:E3224–33. doi: 10.1073/pnas.1323985111, PMID: 25053813 PMC4128120

[37] Phipps JP Haas KM. An adjuvant that increases protective antibody responses to polysaccharide antigens and enables recall responses. J Infect Dis. (2019) 219:323–34. doi: 10.1093/infdis/jiy506, PMID: 30289460 PMC6306023

[38] Gagneux-Brunon A Gagnaire J Pelissier C Berthelot P Botelho-Nevers E. Vaccines for healthcare associated infections without vaccine prevention to date. Vaccine X. (2022) 11:100168. doi: 10.1016/j.jvacx.2022.100168, PMID: 35600984 PMC9118472

[39] Inoue T Kurosaki T. Memory B cells. Nat Rev Immunol. (2023) 24:5–17. doi: 10.1038/s41577-023-00897-3, PMID: 37400644

[40] Dowling DJ van Haren SD Scheid A Bergelson I Kim D Mancuso CJ. TLR7/8 adjuvant overcomes newborn hyporesponsiveness to pneumococcal conjugate vaccine at birth. JCI Insight. (2017) 2:e91020. doi: 10.1172/jci.insight.91020, PMID: 28352660 PMC5360187

[41] Phillips B Van Rompay KKA Rodriguez-Nieves J Lorin C Koutsoukos M Tomai M. Adjuvant-dependent enhancement of HIV Env-specific antibody responses in infant rhesus macaques. J Virol. (2018) 92:e01051–118. doi: 10.1128/JVI.01051-18, PMID: 30089691 PMC6158427

[42] Budroni S Buricchi F Cavallone A Bourguignon P Caubet M Dewar V. Antibody avidity, persistence, and response to antigen recall: comparison of vaccine adjuvants. NPJ Vaccines. (2021) 6:78. doi: 10.1038/s41541-021-00337-0, PMID: 34021167 PMC8140094

[43] Loos C Coccia M Didierlaurent AM Essaghir A Fallon JK Lauffenburger D. Systems serology-based comparison of antibody effector functions induced by adjuvanted vaccines to guide vaccine design. NPJ Vaccines. (2023) 8:34. doi: 10.1038/s41541-023-00613-1, PMID: 36890168 PMC9992919

[44] Givord C Welsby I Detienne S Thomas S Assabban A Lima Silva V. Activation of the endoplasmic reticulum stress sensor IRE1α by the vaccine adjuvant AS03 contributes to its immunostimulatory properties. NPJ Vaccines. (2018) 3:20. doi: 10.1038/s41541-018-0058-4, PMID: 29977610 PMC6023910

[45] Morel S Didierlaurent A Bourguignon P Delhaye S Baras B Jacob V. Adjuvant System AS03 containing α-tocopherol modulates innate immune response and leads to improved adaptive immunity. Vaccine. (2011) 29:2461–73. doi: 10.1016/j.vaccine.2011.01.011, PMID: 21256188

[46] Moris P van der Most R Leroux-Roels I Clement F Dramé M Hanon E. H5N1 influenza vaccine formulated with AS03_A_ induces strong cross-reactive and polyfunctional CD4 T-cell responses. J Clin Immunol. (2011) 31:443–54. doi: 10.1007/s10875-010-9490-6, PMID: 21174144 PMC3132412

[47] Van Damme P Kafeja F Bambure V Hanon E Moris P Roman F. Long-term persistence of humoral and cellular immune responses induced by an AS03_A_-adjuvanted H1N1–2009 influenza vaccine: an open-label, randomized study in adults aged 18–60 years and older. Hum Vaccin Immunother. (2013) 9:1512–22. doi: 10.4161/hv.24504, PMID: 23571166

[48] Galson JD Truck J Kelly DF van der Most R. Investigating the effect of AS03 adjuvant on the plasma cell repertoire following pH1N1 influenza vaccination. Sci Rep. (2016) 6:37229. doi: 10.1038/srep37229, PMID: 27849037 PMC5110968

[49] Cortese M Hagan T Rouphael N Wu SY Xie X Kazmin D. System vaccinology analysis of predictors and mechanisms of antibody response durability to multiple vaccines in humans. Nat Immunol. (2025) 26:116–30. doi: 10.1038/s41590-024-02036-z, PMID: 39747435 PMC12158788

[50] Arunachalam PS Walls AC Golden N Atyeo C Fischinger S Li C. Adjuvanting a subunit COVID-19 vaccine to induce protective immunity. Nature. (2021) 594:253–8. doi: 10.1038/s41586-021-03530-2, PMID: 33873199

[51] Feng Y Yuan M Powers JM Hu M Munt JE Arunachalam PS. Broadly neutralizing antibodies against sarbecoviruses generated by immunization of macaques with an AS03-adjuvanted COVID-19 vaccine. Sci Transl Med. (2023) 15:eadg7404. doi: 10.1126/scitranslmed.adg7404, PMID: 37163615 PMC11032722

[52] Khurana S Coyle EM Manischewitz J King LR Gao J Germain RN. AS03-adjuvanted H5N1 vaccine promotes antibody diversity and affinity maturation, NAI titers, cross-clade H5N1 neutralization, but not H1N1 cross-subtype neutralization. NPJ Vaccines. (2018) 3:40. doi: 10.1038/s41541-018-0076-2, PMID: 30302282 PMC6167326

[53] Leroux-Roels I Borkowski A Vanwolleghem T Drame M Clement F Hons E. Antigen sparing and cross-reactive immunity with an adjuvanted rH5N1 prototype pandemic influenza vaccine: a randomised controlled trial. Lancet. (2007) 370:580–9. doi: 10.1016/S0140-6736(07)61297-5, PMID: 17707753

[54] Goll JB Jain A Jensen TL Assis R Nakajima R Jasinskas A. The antibody landscapes following AS03 and MF59 adjuvanted H5N1 vaccination. NPJ Vaccines. (2022) 7:103. doi: 10.1038/s41541-022-00524-7, PMID: 36042229 PMC9427073

[55] Chen D Wang Y Manakkat Vijay GK Fu S Nash CW Xu D. Coupled analysis of transcriptome and BCR mutations reveals role of OXPHOS in affinity maturation. Nat Immunol. (2021) 22:904–13. doi: 10.1038/s41590-021-00936-y, PMID: 34031613

[56] Luo W Adamska JZ Li C Verma R Liu Q Hagan T. SREBP signaling is essential for effective B cell responses. Nat Immunol. (2023) 24:337–48. doi: 10.1038/s41590-022-01376-y, PMID: 36577930 PMC10928801

[57] Pauksens K Nilsson AC Caubet M Pascal TG Van Belle P Poolman JT. Randomized controlled study of the safety and immunogenicity of pneumococcal vaccine formulations containing PhtD and detoxified pneumolysin with alum or adjuvant system AS02_V_ in elderly adults. Clin Vaccine Immunol. (2014) 21:651–60. doi: 10.1128/CVI.00807-13, PMID: 24599529 PMC4018883

[58] Monaci E Mancini F Lofano G Bacconi M Tavarini S Sammicheli C. MF59- and Al(OH)_3_-adjuvanted *Staphylococcus aureus* (4C-Staph) vaccines induce sustained protective humoral and cellular immune responses, with a critical role for effector CD4 T cells at low antibody titers. Front Immunol. (2015) 6:439. doi: 10.3389/fimmu.2015.00439, PMID: 26441955 PMC4561515

[59] Davis HL. Novel vaccines and adjuvant systems: the utility of animal models for predicting immunogenicity in humans. Hum Vaccin. (2008) 4:246–50. doi: 10.4161/hv.4.3.5318, PMID: 18382138

