# Supplementary material for: Distinct effects of adjuvants on B cell responses to protein or polysaccharide antigens contained in glycoconjugate vaccines
Source: Front Immunol. 2025 Aug 22;16:1574941. doi: 10.3389/fimmu.2025.1574941 (PMC12411546; doi:10.3389/fimmu.2025.1574941)
Supplement: Supplementary Figure 1 — B-cell gating strategies. Flow cytometry gating strategies for phenotypic characterization of antigen-specific B cells in the lymph nodes or spleens (A) or bone marrow (B) are presented. Gating for the antigen-specific populations for immunized mice was selected based on the background derived from phosphate-buffered saline (PBS)-injected mice. (A) Cells were selected based on viability, morphology, and singlets, and B cells were identified as CD19+ cells. Switched (IgD- IgM-) B cells were analyzed for CP5/8 (CP) or Hla positivity. CP+ and Hla+ B cells were further defined by GL7 expression (to identify cells involved in germinal center [GC] reactions) and CD38 expression, which, combined with GL7 downregulation, identified mature B cells about to exit the lymphoid organ. CD38+ GL7- cells were analyzed for CD80 and CD73 expression to identify the mature memory B cells (MBC) that underwent somatic mutation after encountering the antigen. (B) Bone marrow cells were selected based on CD19 expression, viability, morphology, and singlets. Switched (IgD- IgM-) B cells were analyzed for expression of B220 (downregulated in B cells differentiated into plasma cells [PC]) and GL7, to identify B cells involved in GC reactions. B220- GL7- cells were analyzed for CD138 expression to detect PC, and the CD138+ subset was analyzed for antigen positivity. [file Presentation1.pptx]

## Slide 1
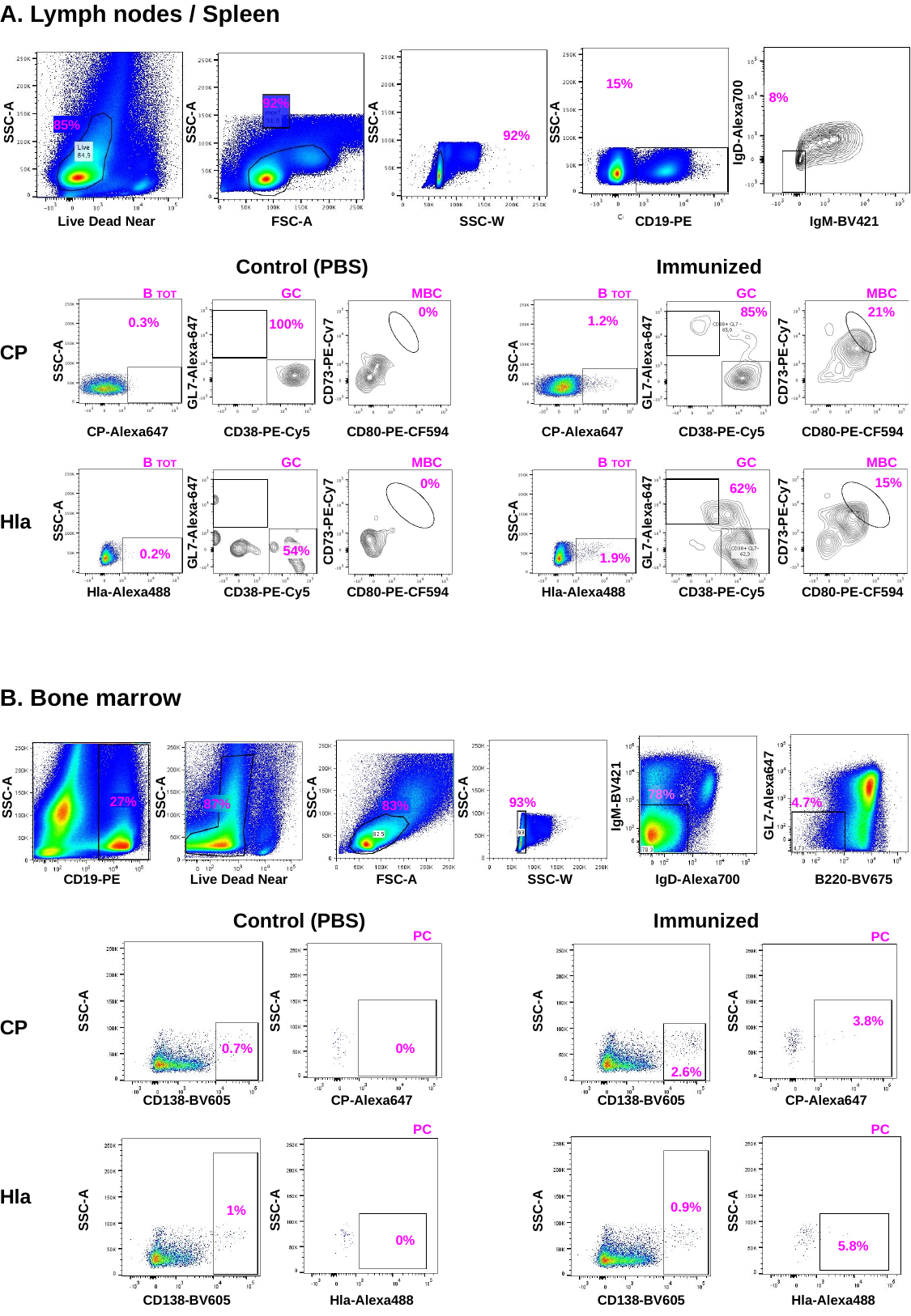

A. Lymph nodes / Spleen
15%
8%
92%
IgD-Alexa700
SSC-A
SSC-A
SSC-A
SSC-A
85%
92%
Live Dead Near
FSC-A
SSC-W
CD19-PE
IgM-BV421
Control (PBS) Immunized
B TOT
GC
MBC
B TOT
GC
MBC
 21%
85%
 0%
1.2%
0.3%
 100%
GL7-Alexa-647
GL7-Alexa-647
SSC-A
SSC-A
CD73-PE-Cy7
CD73-PE-Cy7
CP-Alexa647
CD38-PE-Cy5
CD80-PE-CF594
CP-Alexa647
CD38-PE-Cy5
CD80-PE-CF594
CP
Hla
B TOT
GC
MBC
B TOT
GC
MBC
 15%
 0%
62%
GL7-Alexa-647
GL7-Alexa-647
SSC-A
SSC-A
CD73-PE-Cy7
CD73-PE-Cy7
0.2%
 54%
1.9%
Hla-Alexa488
CD38-PE-Cy5
CD80-PE-CF594
Hla-Alexa488
CD38-PE-Cy5
CD80-PE-CF594
B. Bone marrow
SSC-A
SSC-A
SSC-A
SSC-A
IgM-BV421
GL7-Alexa647
78%
27%
93%
4.7%
87%
 83%
CD19-PE
Live Dead Near
FSC-A
SSC-W
IgD-Alexa700
B220-BV675
Control (PBS) Immunized
PC
PC
SSC-A
SSC-A
SSC-A
SSC-A
3.8%
CP
Hla
0%
0.7%
2.6%
CD138-BV605
CP-Alexa647
CP-Alexa647
CD138-BV605
PC
PC
0.9%
SSC-A
SSC-A
SSC-A
SSC-A
1%
0%
5.8%
CD138-BV605
Hla-Alexa488
Hla-Alexa488
CD138-BV605
