# Supplementary figures and images for: Distinct effects of adjuvants on B cell responses to protein or polysaccharide antigens contained in glycoconjugate vaccines
Source: Front Immunol. 2025 Aug 22;16:1574941. doi: 10.3389/fimmu.2025.1574941 (PMC12411546; doi:10.3389/fimmu.2025.1574941)

## Slide 1
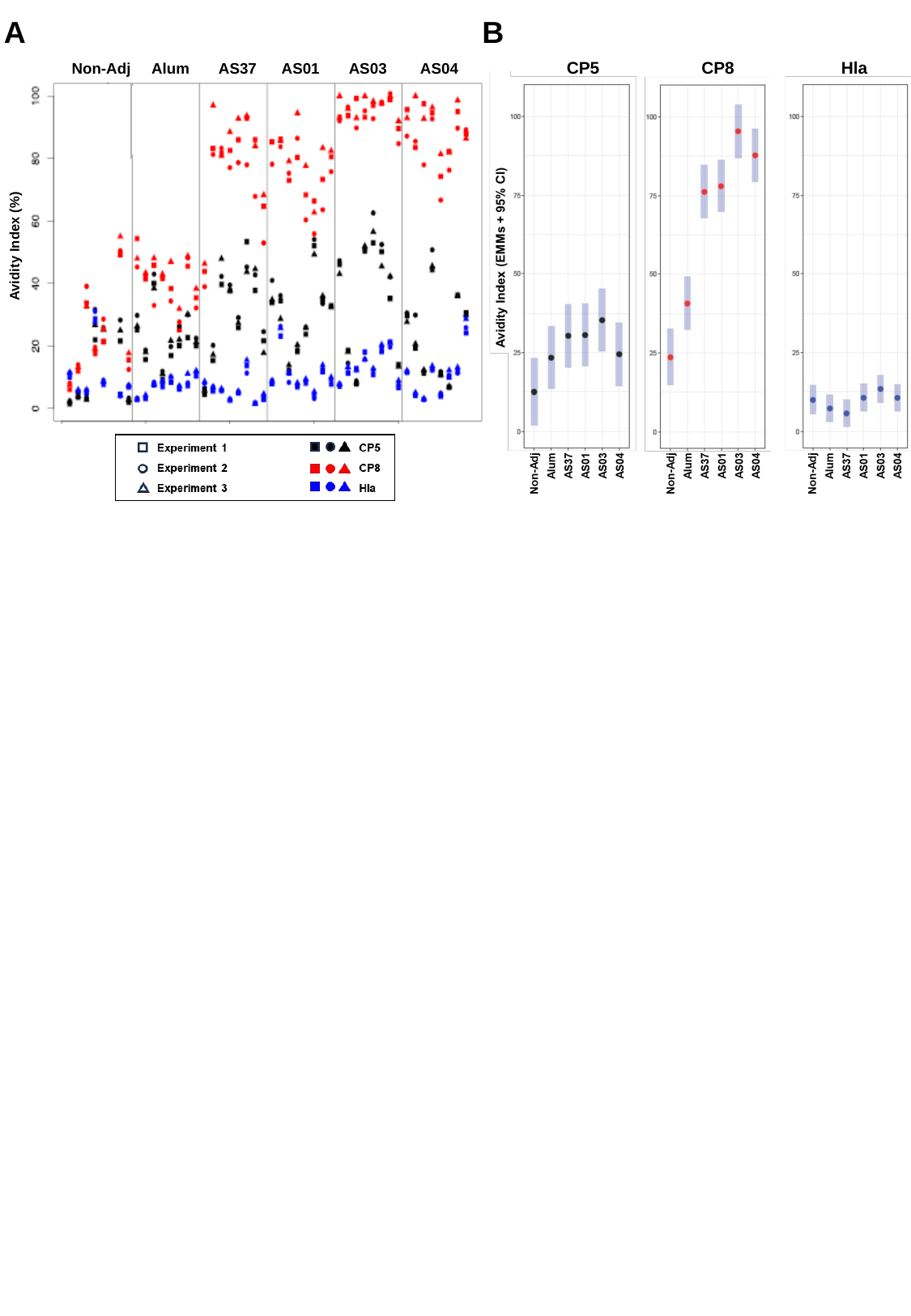

A B
CP5 CP8 Hla
Non-Adj Alum AS37 AS01 AS03 AS04
Avidity Index (%)

Supplement: Supplementary Figure 2 — Antibody avidity preliminary study. Mice (n=8/group) received two intramuscular injections, four weeks apart, of a CP5-TT/CP8-TT/Hla vaccine that was either adjuvanted (with AS01, AS03, AS04, AS37, or Alum), or non-adjuvanted. Blood samples were collected two weeks after the second dose. (A) The avidity indices of antigen-specific antibodies of each individual serum sample were measured in three separate experiments. Each symbol represents a single measurement for one individual animal. (B). Estimated marginal means (EMMs; colored dots) of the avidity indices derived from a mixed effect model are presented with 95% confidence intervals (CI; grey vertical bars). [file Presentation2.pptx]
