# Supplementary material for: Distinct effects of adjuvants on B cell responses to protein or polysaccharide antigens contained in glycoconjugate vaccines
Source: Front Immunol. 2025 Aug 22;16:1574941. doi: 10.3389/fimmu.2025.1574941 (PMC12411546; doi:10.3389/fimmu.2025.1574941)
Supplement: Supplementary Figure 3 — Antigen-specific B-cell expansion in the bone marrow. (A) Schedule shows the timepoints of the treatments with Staphylococcus aureus vaccines containing non-adjuvanted and adjuvanted CP5, CP8, and Hla antigens (bracketed) and tissue collection timepoints of the bone marrow samples at 9, 17, and 25 weeks post-dose 3 (pIII). Mice received three intramuscular injections of CP5-TT/CP8-TT/Hla vaccine either adjuvanted with Alum, AS01, AS03, AS04 or AS37, or non-adjuvanted. A control group received phosphate-buffered saline (PBS). For each group, samples from 5 mice were combined into a single pool. (B) Numbers of antigen-specific long lived plasma cells/million singlets are presented as a single data point per group, color-coded as shown in the key. [file Presentation3.pptx]

## Slide 1
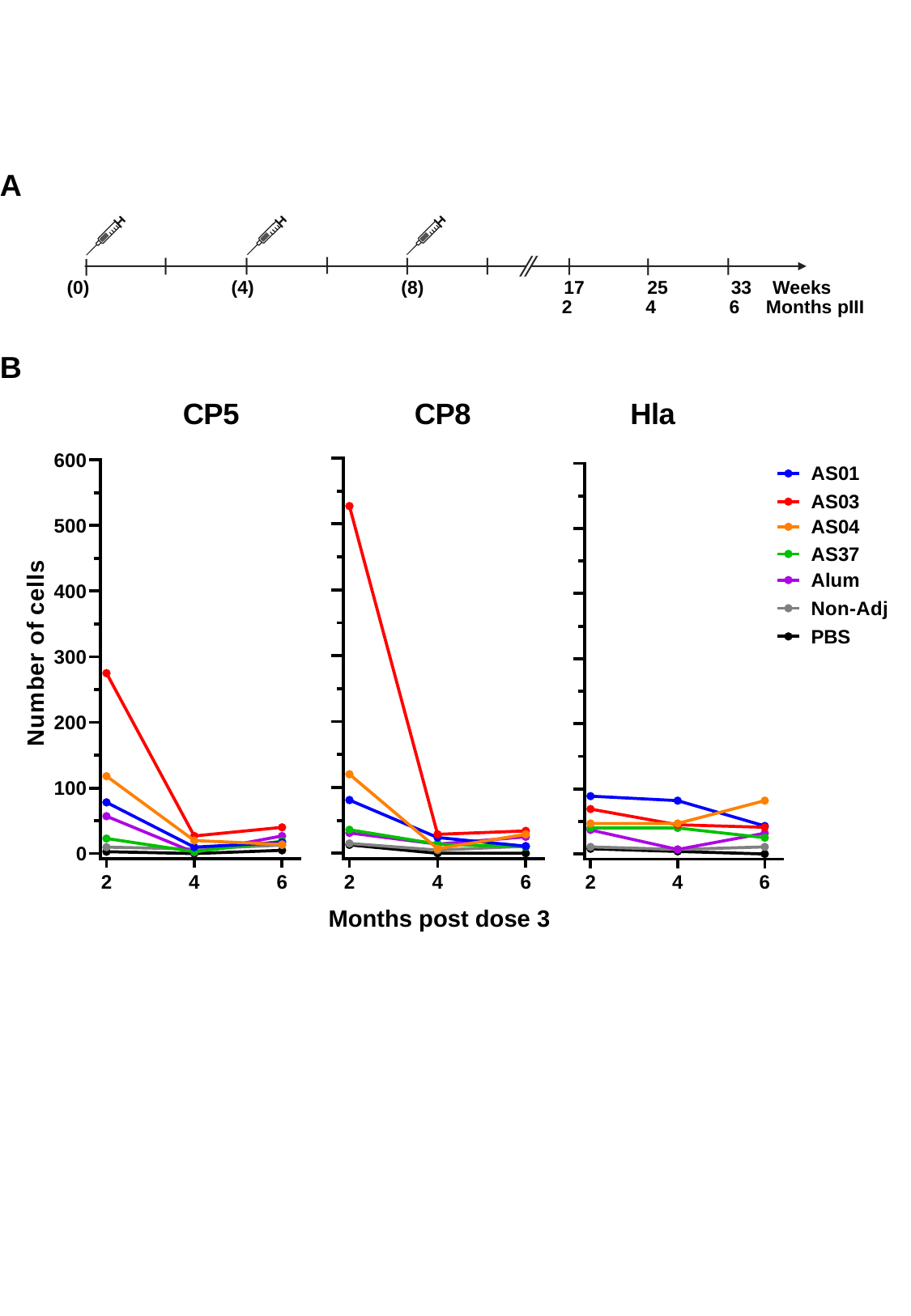

Data file: D119, D179, D231
Report: D117, D123, D183 on page 8
 But D119, D179, D231 on page 25 = W17, W25, W33, or W9, W17, W25 pIII/M2, M4, M6 pIII
A
B
(0) (4) (8) 17 25 33 Weeks
2 4 6 Months pIII
Months post dose 3
