# Supplementary material for: Distinct effects of adjuvants on B cell responses to protein or polysaccharide antigens contained in glycoconjugate vaccines
Source: Front Immunol. 2025 Aug 22;16:1574941. doi: 10.3389/fimmu.2025.1574941 (PMC12411546; doi:10.3389/fimmu.2025.1574941)
Supplement: Supplementary Table 1 — Design adjuvant dose-finding experiments. *A/C, active/control groups. **volume-based ratios for 25 μL AS03 + 25 μL antigen mixture. Bold font, dose that was ultimately selected for further experiments. Alum, aluminum hydroxide. AS37 contained a synthetic TLR7 agonist (TLR-7a) adsorbed on Alum. [file SupplementaryFile1.docx]

**Supplementary methods**

**S1 Studies and ethics**

All studies were performed in GSK’s AAALAC-accredited animal facilities in Siena, Italy. Husbandry/experiments were ethically reviewed and performed in accordance with Italian and European laws/guidelines/policies for animal experimentation, housing, and care (Italian D. L. no. 26/14 and the European Directive 2010/63/UE) and GSK’s Policy on the Care, Welfare, and Treatment of Animals. Protocols for all studies were approved by the local ethical review committees of GSK (reference AWB2015-01).

**S2 Adjuvant dose-ranging experiments**

As used in the main study, the SA glycoconjugate study vaccines contained the mutant inactivated form of SA-Hla toxin (HlaH35L; ‘Hla’) and the SA CP5 and CP8 conjugated with tetanus toxoid (CP5-TT and CD8-TT, respectively).

The objective was to identify the optimal adjuvant doses for the induction of anti-CP5/8 antibody responses for use in subsequent studies. Four (AS01/AS03/Alum) or five (AS04/AS37) different doses per adjuvant were evaluated in three separate experiments. Each experiment evaluated two adjuvants (AS01/AS03, Alum/AS37 or Alum/AS04) in different doses, and included control groups receiving phosphate-buffered saline (PBS) with or without antigen, or adjuvant only (see **Table S1** below).

A total of 280 female BALB/c mice (Charles River; n=10/group) received two intramuscular injections of 50 μL (25μL per leg), 4 weeks apart. The antigen mixture per dose contained 10 μg Hla and 10 μg (based on TT concentration) each of TT-CP5 and TT-CP8 in PBS. Blood samples were collected at days 14 and 42 (14 days post I and II, respectively). Animal husbandry, blood sample collection, and assays to measure binding antibody responses were similar to those in the main study.

Data were statistically analyzed by timepoint/antigen combination, as follows: ANOVA models were fitted on log10 antibody titers (assuming heterogeneous variances between groups), and were used to estimate geometric mean titers (GMTs) with 95% confidence intervals (CIs) and geometric mean ratios (GMRs, with 95% CIs) per active group vs the corresponding antigen-only group.

Across the antigens, most of the results showed that adjuvant dose-dependency of the antibody titers was present for each adjuvant (data not shown). Therefore, the highest adjuvant doses tested (indicated in Table S1 below) were selected for further evaluation in the follow-up studies (a preliminary study, as described below, and the main study).

**S3 Preliminary study**

A total of 199 female BALB/c mice (Charles River) were used. Seven groups (n=28/group) received two intramuscular injections of 50 μL (25μL per leg), 4 weeks apart, and a control group (n=3) were left untreated. Six groups were vaccinated with the antigen mixture (10 μg Hla and 2 μg [based on TT concentration] each of TT-CP5 and TT-CP8) combined with either an adjuvant (AS01, AS03, AS04, AS37, or Alum) at the selected dose (see Table S1) or with PBS. A seventh group received PBS only.

Antibody avidity was measured two weeks after the first or second immunization (n=24 or n=8 respectively). Data from each mouse were tested in three independent experiments. Animal husbandry, assays, and statistical analyses were performed as described for the main study.

**Table S1. Design adjuvant dose-finding experiments**

| **Exp.** | **Adjuvant** | **Adjuvant dose (per 50 μL injection)** | **+/-Antigen mixture** | **A / CA/C group*** |
| --- | --- | --- | --- | --- |
| **1** | AS01 | **2.5 μg MPL + 2.5 μg QS-21 + liposomes** | + | A |
|  |  | 1.25 μg + 1.25 μg QS-21 + liposomes | + | A |
|  |  | 0.4 μg + 0.4 μg QS-21 + liposomes | + | A |
|  |  | 2.5 μg MPL + 2.5 μg QS-21 + liposomes | - | C |
|  | - | 0 μg (PBS) | + | C |
|  | AS03 | **1:1 v/v**** | + | A |
|  |  | 1:5 v/v** | + | A |
|  |  | 1:10 v/v** | + | A |
|  |  | (AS03 only) | - | C |
|  | - | - (PBS) | - | C |
| **2** | AS37 | **20 μg TLR-7a in 2 mg/mL Alum** | + | A |
|  |  | 10 μg TLR-7a in 2 mg/mL Alum | + | A |
|  |  | 10 μg TLR-7a in 1 mg/mL Alum | + | A |
|  |  | 2 μg TLR-7a in 2 mg/mL Alum | + | A |
|  |  | 2 μg TLR-7a in 0.2 mg/mL Alum | + | A |
|  | Alum | **2 mg/mL** | + | A |
|  |  | 1 mg/mL | + | A |
|  |  | 0.2 mg/mL | + | A |
|  | - | 0 mg/mL (PBS) | + | C |
| **3** | AS04 | **10 μg MPL in 1 mg/mL Alum** | + | A |
|  |  | 5 μg MPL in 1 mg/mL Alum | + | A |
|  |  | 5 μg MPL in 0.5 mg/mL Alum | + | A |
|  |  | 2 μg MPL in 1 mg/mL Alum | + | A |
|  |  | 2 μg MPL in 0.2 mg/mL Alum | + | A |
|  | Alum | 1 mg/mL | + | A |
|  |  | 0.5 mg/mL | + | A |
|  |  | 0.2 mg/mL | + | A |
|  | - | 0 mg/mL (PBS) | + | C |

*A/C, active/control groups. **volume-based ratios for 25 μL AS03 + 25 μL antigen mixture. Bold font, dose that was ultimately selected for further experiments. Alum, aluminum hydroxide. AS37 contained a synthetic TLR7 agonist (TLR-7a) adsorbed on Alum.
